# Data on a study of the challenges faced by parents of special needs children and their impact on parental health and well-being in Trinidad and Tobago: A qualitative study

**DOI:** 10.1016/j.dib.2026.113184

**Published:** 2026-08-22

**Authors:** Georgelle Da Briel, Gabrielle De Bique, Hasina Logie, Ian Mahabir, Imojin Mayers, Hannah Mendoza, Ishana Sirju, Marcia Nathai-Balkissoon

**Affiliations:** a School of Medicine, Faculty of Medical Sciences, The University of the West Indies, Trinidad and Tobago; b Department of Management Studies, Faculty of Social Sciences, The University of the West Indies, Trinidad and Tobago

**Keywords:** Caregiver stress, Emotional burden, Social support, Health impact, Qualitative interviews, Family dynamics

## Abstract

This article presents a qualitative interview dataset collected in Trinidad and Tobago, focusing on the lived experiences of parents of children with special needs. The dataset captures rich, first-hand accounts of the emotional, social, and practical challenges encountered by parents, as well as their interactions with local healthcare, educational, and social support systems. Ethical approval was obtained from the Campus Research Ethics Committee, The University of the West Indies, St. Augustine, and all participants provided informed verbal consent before participation.

Data were collected through semi-structured one-on-one interviews conducted virtually via Zoom to ensure accessibility, confidentiality, and participant comfort. Although participants were given the option of in-person interviews, all opted for virtual participation. Recruitment was facilitated through advocacy and community organizations supporting special needs families. Purposive and snowball sampling methods were employed to select 15 participants who met the inclusion criteria of being parents of special needs dependents. The participants varied across several demographic characteristics. Their ages ranged from 31 to 69 years. Marital status included single, married, and widowed individuals. Participants had between one and four children, with some having one or two children with special needs. Educational attainment ranged from secondary-level education to tertiary-level education. Both male and female participants were represented. All interviews were transcribed verbatim and anonymized before analysis. Thematic analysis was performed using QDA Miner software through a multistage process involving data coding, categorization, and synthesis. Data saturation determined the final sample size, ensuring adequate depth and diversity across socioeconomic, geographic, and disability type variations.

The dataset includes anonymized interview transcripts and coded output tables produced during thematic analysis. It provides detailed qualitative evidence on caregiving practices, systemic barriers, and psychosocial impacts experienced by parents of special needs children in a small-island developing state. The dataset offers strong potential for reuse in cross-cultural or longitudinal analyses on caregiver well-being, disability inclusion, and social support mechanisms, as well as for informing evidence-based policy development and program design in similar regional contexts.

Specifications Table

**Table:** 

Subject	Please select Subject from the dropdown list.
Specific subject area	ChallengesandHealthImpactsofParentingChildrenwithSpecialNeeds
Type of data	Raw Text excerpts, Coded Data, Analyzed, Themed categories. Tables
Data collection	Data were collected through semi-structured one-on-one interviews conducted via Zoom using an interview guide developed by the researchers. Participants were parents of special needs children in Trinidad and Tobago, selected through purposive and snowball sampling. Inclusion required active caregiving; non-caregivers were excluded. Interviews were audio-recorded, transcribed verbatim, anonymized, and analyzed thematically using QDA Miner software.
Data source location	The data were collected at The UWI, St. Augustine Campus, Trinidad and Tobago.
Data accessibility	Repository name: Mendeley Data Data identification number: 10.17632/bd9bk8cjg7.3 Direct URL to data: https://data.mendeley.com/datasets/bd9bk8cjg7/4
Related research article	Georgelle Da Briel, Gabrielle De Bique, Hasina Logie, Ian Mahabir, Imojin Mayers, Hannah Mendoza, Ishana Sirju, Marcia Nathai-Balkissoon. A Study of the Challenges Faced by Parents of Special Needs Children and Their Impact on Health and Well-Being: A Qualitative Study. (Under Review.) [1].

## 1 Value of the Data

• These data provide in-depth qualitative insights into the lived experiences of parents of special needs children in Trinidad and Tobago, highlighting the physical, mental, emotional, social, and financial challenges they face.
• The data captures local realities of healthcare access, financial strain, and service/support inadequacies. This offers a deeper understanding of gaps affecting parental health and well-being.
• These data offer context-rich narratives that can be reused to explore intersections between disability, gender roles, socioeconomic status, and mental health within Caribbean, small-island states, or developing-country contexts.
• The data can support future studies examining policy gaps, disability inclusion, and the effectiveness of national support systems, providing a foundation for comparative or longitudinal public health or social research.
• The structured coding and thematic analysis using QDA Miner can support qualitative data organization and secondary analysis in similar small-sample qualitative studies.

## 2 Background

Existing literature shows that parents of children with disabilities face elevated stress, financial strain, and emotional exhaustion, but research on these experiences in Trinidad and Tobago and the wider Caribbean remains limited. Richards [2] found no research on the experiences of parents of children with autism spectrum disorder in Trinidad and Tobago, while Taklalsingh [3] highlighted the broader lack of local research on parents of children with disabilities, underscoring the need for context-specific evidence to inform policy, support services, and public awareness [4,5] This dataset was compiled to document the challenges experienced by parents raising children with special needs in Trinidad and Tobago and to understand how these lived experiences influence their physical and mental well-being.

This study was motivated by the need to examine systemic and personal challenges affecting parent-caregivers’ health and well-being, including limited access to specialized services, low public awareness, and the emotional burden of raising a child with special needs. Using semi-structured interviews and thematic analysis, the study prioritized participants’ voices, capturing the meaning and quality of their experiences [5]. This dataset complements future research articles by providing the full anonymized transcripts, thematic analyses, and code tables that underpin the published findings, offering transparency and the potential for secondary qualitative analysis.

## 3 Data Description

The dataset contains qualitative interview data obtained from parents who were the primary caregivers of their own child or adult dependents with special needs in Trinidad and Tobago. The dataset serves to document the lived experiences, challenges, and coping mechanisms of caregivers in a local social, health, and educational context.

All interviews were conducted virtually and transcribed verbatim. Each transcript was anonymised to remove personal identifiers such as names, locations, and institutions. The dataset is provided in multiple formats to support transparency and reusability.

The repository includes the following components:

• Text excerpts.pdf - This part of the document contains phrases of text taken from the transcripts of the participants.
• Coded Data.pdf– This part of the document contains the text excerpts encoded.
• Themes Summary.pdf – This part of the document outlines the main themes that emerged during the coding process, such as parent stress, emotional and physical well-being, social support, access to services, and financial strain.
• Interview Guide.pdf – The semi-structured interview guide used during data collection, which details the major discussion areas and question prompts.
• Consent.pdf – A document describing the consent process.
• Sociodemographic information of participants - A document describing participants' age, marital status, number of children, number of special needs children, and type of special need the children have.

The dataset provides detailed qualitative insights into special needs parenting experiences within Trinidad and Tobago. Each transcript captures multiple dimensions of the parents’ realities, including emotional challenges, support systems, and interactions with healthcare and educational institutions. The coding files support secondary analyses by enabling researchers to re-examine or expand upon the thematic framework applied.

The data files are presented in open and accessible formats (PDF) and can be used for cross-cultural comparisons, methodological replication, or further thematic studies on caregiving, disability, or social support in small-island contexts. No identifying information is included, and all data were collected under ethical approval from the University of the West Indies Ethics Committee and the Ministry of Health of Trinidad and Tobago.

## 4 Experimental Design, Materials and Methods

### 4.1 Data acquisition/methods

The dataset was compiled from a qualitative study conducted in Trinidad and Tobago, using semi-structured interviews to explore the experiences of parents who were primary caregivers of their child or adult dependents with special needs. Data collection focused on capturing participants’ perspectives on the challenges they faced, as well as the effects on their physical and mental well-being.

### 4.2 Participant recruitment and inclusion criteria

Participants were recruited through purposive and snowball sampling via community organizations and advocacy groups supporting parents of children with special needs. Inclusion criteria required participants to be parents actively involved in raising children with special needs, including adult dependents. Individuals not meeting these criteria were excluded. A total of 15 participants were included. This sample size is consistent with recommendations for qualitative research such as phenomenological studies (if done on more than one person), where relatively small, information-rich samples are considered appropriate to facilitate in-depth exploration of participants' experiences and achieve data saturation [[6], [7], [8]]. A total of 15 participants were included. After the initial 12 interviews, three additional interviews contributed further examples and nuance but did not materially alter the developing coding and category structure, indicating that the stage 1 coding/category structure had stabilised by the end of data collection.

### 4.3 Data collection

Data were collected using semi-structured interviews conducted one-on-one via Zoom. Virtual interviews were chosen to maximize accessibility, confidentiality, and participant convenience; although in-person interviews were offered, all participants opted for virtual participation. Interviews were audio-recorded with participants’ consent. Verbal informed consent was obtained and recorded prior to each session. Sessions lasted between 14 min and 2 h and 31 min.

The interview guide used was developed by the researchers and contained open-ended questions to capture experiences, challenges, coping strategies and recommendations, and perceived impacts on health and well-being. The guide was pilot-tested with a small group of 3 parents, and adjusted to ensure clarity, relevance, and accuracy in the themes addressed, wording and comprehensibility of questions, and organisation of sections and flow for the interview before being used for data collection. There was no overlap between the participants used in the pilot exercise and the participants of the study.

### 4.4 Data management and transcription

Audio recordings were transcribed verbatim and anonymized to remove any personal identifiers. Transcripts were stored as .txt files and organized in a structured folder.

### 4.5 Data analysis

Transcripts were analyzed using QDA Miner (Provalis Research, version 6). The analysis procedure included:

- Importing transcripts into QDA Miner.
- Identifying relevant text excerpts and applying codes according to content similarity.
- Grouping codes into categories and subthemes to identify patterns across participants.
- Generating tables of coded excerpts and thematic categories exported as .xlsx and .csv files.
- Synthesizing coded data to produce integrated summaries for each research question, saved as .docx.

### 4.6 Dataset structure

The dataset is organized as follows:

- Text excerpts: Table format, Coded Data: QDA Miner coding tables, Analysis Outputs: Theme summary tables (.pdf)
- Documentation : consent form and interview guide(.pdf), sociodemographic information of participants (.pdf)

All files are in open formats to facilitate reuse for secondary qualitative analysis, methodological studies, or comparative research.

## Limitations

This qualitative study focused on depth rather than breadth, so findings may not be generalizable to all special needs parents in Trinidad and Tobago. The sample comprised 15 participants, and was intended to provide in-depth, context-specific qualitative accounts. Nevertheless, the relatively small sample may limit the transferability of the findings to other populations and settings. Using purposive and snowball sampling introduced potential selection bias, as many participants were active in advocacy or support groups, possibly excluding more isolated caregivers. Efforts were made to reduce this by screening parents who had children with various disabilities. Data collected through semi-structured Zoom interviews were subject to recall, personal, and social desirability bias, given the sensitive nature of the topic. Interviewers minimized this possible omission of pertinent details by asking follow-up questions. Conducting interviews using Zoom offered convenience but technical challenges such as poor internet connectivity occasionally disrupted discussions, though questions were repeated and clarified or repeated responses requested, so that there was no loss of data on which the analysis would depend.

## Ethics Statement

This study was conducted in accordance with the ethical standards of the University of the West Indies (UWI) Ethics Committee (Approval number: CREC-SA.2988/11/2024) and the Ministry of Health of Trinidad and Tobago and with the principles outlined in the Declaration of Helsinki (revised 2013). Ethical approval for the research protocol was obtained from the UWI Ethics Committee and the Ministry of Health of Trinidad and Tobago.

All participants were informed about the purpose and procedures of the study, and verbal consent was obtained from each participant prior to data collection. Participation was voluntary, and participants were assured of confidentiality and the right to withdraw from the study at any time without penalty.

A copy of the informed consent form (in English) is available in the Mendeley Data submission.

## CRediT Author Statement

**Georgelle Da Briel:** Conceptualization, Investigation, Visualization, Writing- Review and Editing, Methodology; **Gabrielle De Bique:** Conceptualization, Investigation, Project administration, Visualization, Writing- Review and Editing, Methodology; **Hasina Logie:** Conceptualization, Investigation, Data Curation, Project administration, Resources, Writing- Review and Editing, Methodology; **Ian Mahabir:** Conceptualization, Investigation, Writing- Review and Editing, Visualization, Methodology; **Imojin Mayers:** Conceptualization, Investigation, Project administration, Visualization, Writing- Review and Editing, Methodology; **Hannah Mendoza:** Conceptualization, Investigation, Visualization, Writing- Review and Editing, Methodology; **Ishana Sirju:** Conceptualization, Investigation, Writing- Review and Editing, Visualization, Methodology; **Marcia Nathai-Balkissoon:** Conceptualization, Methodology, Validation, Formal Analysis, Resources, Writing - Review and Editing, Visualization, Supervision, Project administration.

## Data Availability

Data on a study of the challenges faced by parents of special needs children and their impact on parental health and well-being: A Qualitative Study (Original data). Mendeley DataSpecialNeedsParents_TT_Data (Original data).
